# Highly Pathogenic Avian Influenza A(H5N1) Clade 2.3.4.4b Virus Infection in Poultry Farm Workers, Washington, USA, 2024

**DOI:** 10.3201/eid3112.251118

**Published:** 2025-12

**Authors:** Yasuko Hatta, Juan A. De La Cruz, Theresa Murray, Brian Hiatt, Yunho Jang, Julia C. Frederick, Kristine A. Lacek, Juliana C. DaSilva, Dan Cui, Paul Carney, Jimma Liddell, Kay W. Radford, Natasha Burnett, Sabrina Schatzman, Pauline Trinh, Anna Unutzer, Elizabeth A. Pusch, Monique Johnson, Ha T. Nguyen, Benjamin L. Rambo-Martin, Larisa Gubareva, James Stevens, C. Todd Davis, Marie K. Kirby, Allison Black, Han Di

**Affiliations:** Centers for Disease Control and Prevention, Atlanta, Georgia, USA (Y. Hatta, J.A. De La Cruz, Y. Jang, J.C. Frederick, K.A. Lacek, J.C. DaSilva, D. Cui, P. Carney, J. Liddell, K.W. Radford, N. Burnett, S. Schatzman, E.A. Pusch, M. Johnson, H.T. Nguyen, B.L. Rambo-Martin, L. Gubareva, J. Stevens, C.T. Davis, M.K. Kirby, H. Di); Washington State Department of Health, Shoreline, Washington, USA (T. Murray, B. Hiatt, P. Trinh, A. Unutzer, A. Black)

**Keywords:** Influenza, viruses, zoonoses, respiratory infections, avian influenza A virus, H5N1, genotype D1.1, receptor binding, candidate vaccine virus, antigenicity, Washington, United States

## Abstract

Poultry workers in Washington, USA, were infected with highly pathogenic avian influenza A(H5N1) virus and recovered. The viruses were clade 2.3.4.4b genotype D1.1, closely related to viruses causing poultry outbreaks. Continued surveillance and testing for influenza A(H5) clade 2.3.4.4b viruses remain essential for risk assessment and pandemic preparedness of zoonotic influenza viruses.

The global spread of A/goose/Guangdong/96-lineage highly pathogenic avian influenza (HPAI) virus of the A(H5) subtype has resulted in numerous clades, subclades, and genotypes because of continuous genetic drift and reassortment. HPAI H5N1 clade 2.3.4.4b virus is the most widespread globally; since December 2021, that clade has circulated in wild birds in the United States, affecting millions of poultry, mammalian wildlife, domestic livestock, and companion animals (*1*,*2*). In 2024, two distinct genotypes were mainly responsible for ongoing outbreaks in the United States, B3.13 mostly in dairy cattle and D1.1 in poultry, but outbreaks of both genotypes were reported in cattle and poultry (*3*,*4*). 

Sporadic human infections with clade 2.3.4.4b viruses have also been reported in the United States. During April 1, 2024–June 30, 2025, a total of 70 human cases were reported, including 41 cases after dairy cattle exposure, 24 after exposure to commercial poultry flocks, 2 after exposure to backyard flocks, and 3 with an unknown exposure source (*5*,*6*). In response to reported human infections with clade 2.3.4.4b viruses in the United States and other countries, several 2.3.4.4b A(H5) prepandemic candidate vaccine viruses (CVVs) have been made available for pandemic influenza preparedness (*7*). 

In late 2024, the Washington State Public Health Laboratory detected influenza A(H5) virus by real-time reverse transcription PCR among specimens from poultry workers experiencing influenza symptoms. We investigated virus isolates from human cases in Washington to determine virus receptor-binding preference and cross-reactivity with existing CVVs.

## The Study

During October 23–November 5, 2024, the Centers for Disease Control and Prevention (CDC; Atlanta, GA, USA) received multiple presumptive influenza A(H5)–positive human clinical specimens from the Washington State Public Health Laboratory. Testing at CDC confirmed HPAI A(H5) virus in 8 poultry farm workers. All 8 cases occurred in adults exposed to H5N1 virus–infected poultry during depopulation efforts to control an outbreak among poultry in Washington. Each affected person reported conjunctivitis, and some also reported respiratory symptoms (*6*). The mean cycle threshold (Ct) value to detect influenza A matrix gene from the H5-positive specimens was 33 (range 25–36.9). 

After multiple genetic sequencing attempts at CDC, we obtained complete hemagglutinin (HA) gene sequences from 5 confirmed cases and partial or complete neuraminidase (NA) gene sequences from 4 confirmed cases. On the basis of the available HA and NA sequences, the viruses in the specimens belonged to HPAI H5N1 clade 2.3.4.4b. Sequences of the internal gene segments were only available from 4 human cases at various levels of completion. We observed minimal genetic variation among human cases; we submitted all available gene sequences to GISAID (https://www.gisaid.org) and GenBank (Appendix Table 1). 

We attempted virus isolation by inoculating the positive specimens in 10–11-day-old embryonated chicken eggs, MDCK cells, or both. We isolated A/Washington/239/2024 from a conjunctival specimen in MDCK cells and isolated A/Washington/254/2024 and A/Washington/255/2024 from the conjunctival specimens in eggs. We also isolated A/Washington/240/2024 in eggs from both conjunctival and nasopharyngeal specimens (Table).

**Table:** Hemagglutination inhibition testing of highly pathogenic avian influenza A(H5N1) clade 2.3.4.4b genotype D1.1 viruses isolated from poultry farm workers, Washington, USA, 2024*

Antigens	Subtype	Clade	Hemagglutination inhibition titer†						
			CDC-RG	RG71A	RG71A	RG78A	RG78A	NIID-002	TX/37
Reference antigens									
CDC-RG, A/Vietnam/1203/2004	H5N1	1	**2,560**	<10	<10	<10	<10	<10	<10
IDCDC-RG71A, A/Astrakhan/3212/2020	H5N8	2.3.4.4b	10	**160**	**160**	80	80	40	<10
IDCDC-RG78A, A/American Wigeon/ South Carolina/22-000345-001/2021	H5N1	2.3.4.4b	10	160	160	**160**	**320**	80	80
NIID-002, A/Ezo red fox/Hokkaido/1/2022	H5N1	2.3.4.4b	10	160	320	160	160	**80**	80
A/Texas/37/2024	H5N1	2.3.4.4b	10	80	80	160	160	80	**80**
Test antigens									
A/Washington/239/2024, conjunctival‡	H5N1	2.3.4.4b	10	80	160	160	160	80	80
A/Washington/240/2024, conjunctival§	H5N1	2.3.4.4b	10	80	80	160	160	40	40
A/Washington/240/2024, nasopharyngeal§	H5N1	2.3.4.4b	10	80	80	320	320	80	80
A/Washington/254/2024, conjunctival§	H5N1	2.3.4.4b	10	80	40	160	160	80	80
A/Washington/255/2024, conjunctival§	H5N1	2.3.4.4b	10	40	40	160	160	80	40

*Bold indicates homologous titers. TX/37, A/Texas/37/2024. †Two different lots of ferret antiserum against RG71A or RG78A were used in this test. ‡Isolated on MDCK cells. §Isolated in 10–11-day-old embryonated chicken eggs.

**Figure 1:**
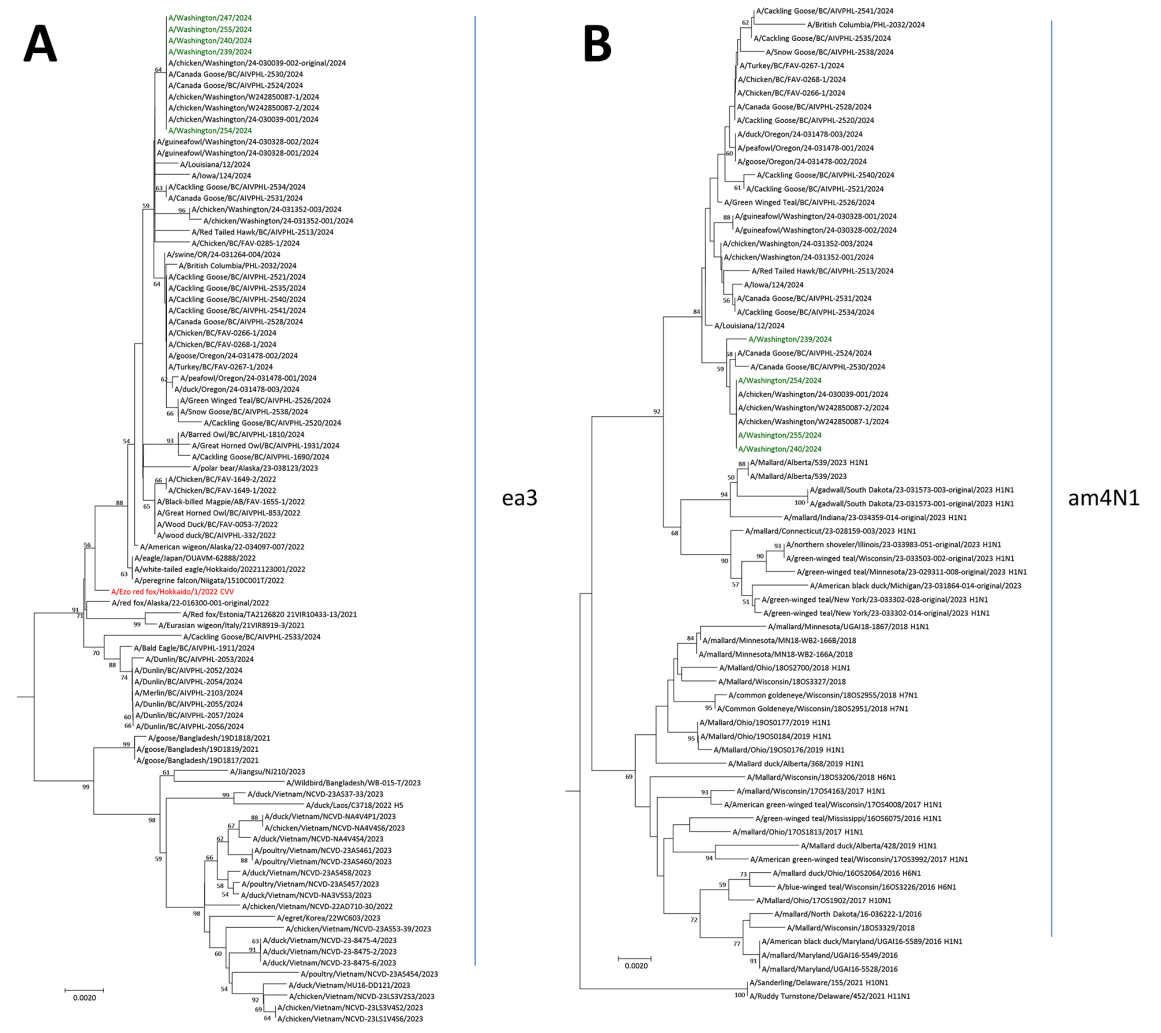
Neighbor-joining phylogenetic trees of highly pathogenic avian influenza A(H5N1) clade 2.3.4.4b viruses isolated from poultry farm workers, Washington, USA, 2024. A) Hemagglutinin gene segment; B) neuraminidase gene segment. Green font indicates influenza A(H5N1) D1.1 viruses from Washington human cases; red font indicates prepandemic candidate vaccine virus A/Ezo red fox/Hokkaido/1/2022. Bootstrap values >50, generated from 1,000 replicates, are labeled on branch nodes. Scale bars indicate nucleotide substitutions per site.

Since late 2021, at least 6 distinct introductions of clade 2.3.4.4b A(H5) viruses from Europe and Asia (genotypes A1–A6) have occurred in the United States. Each introduction was followed by reassortment events that generated many different genotypes (*8*,*9*). Phylogenetic analysis of the 8 gene segments from H5N1 viruses in the specimens of the Washington human cases determined that they belonged to genotype D1.1. That genotype derived from the Eurasian genotype A3 and acquired North American wild bird lineage polymerase basic (PB) 2 (am24 group), polymerase acidic (am4 group), nucleoprotein (am13 group), and NA (am4N1 group) gene segments (Figure 1; Appendix Figures 1–6). Genotype D1.1 is different from genotype B3.13, which derived from the Eurasian genotype A1 after acquiring North American wild bird lineage PB2 (am2.2 group), PB1 (am4 group), nucleoprotein (am8 group), and nonstructural (am1.1 group) gene segments after reassortment. 

Phylogenetically, HA sequences from the Washington human cases belonged to the Eurasian ea3 group and were closely related to viruses detected from the Washington poultry outbreak. Those HA sequences also resembled sequences from wild birds detected in British Columbia, Canada, during 2024 (Figure 1, panel A). The HA sequences from the Washington D1.1 human cases did not contain mutations known to be associated with increased infectivity or transmissibility among humans (FluSurver, https://flusurver.bii.a-star.edu.sg). The NA gene segments of H5N1 clade 2.3.4.4b viruses circulating in the United States have been predominantly Eurasian lineage since their introduction (*8*). However, the D1.1 viruses detected in the Washington cases all contained North American lineage N1 NA genes closely related to those of H5N1 viruses detected in poultry and wild birds in British Columbia in 2024 and H1N1 viruses detected in wild birds in the United States and Canada in 2023 (Figure 1, panel B). The available NA and internal gene sequences from the Washington D1.1 human cases lacked changes associated with reduced antiviral susceptibility or mammalian adaptation. They also lacked PB2-M631L mutation that was detected in most B3.13 viral sequences (*10*–*12*).

**Figure 2:**
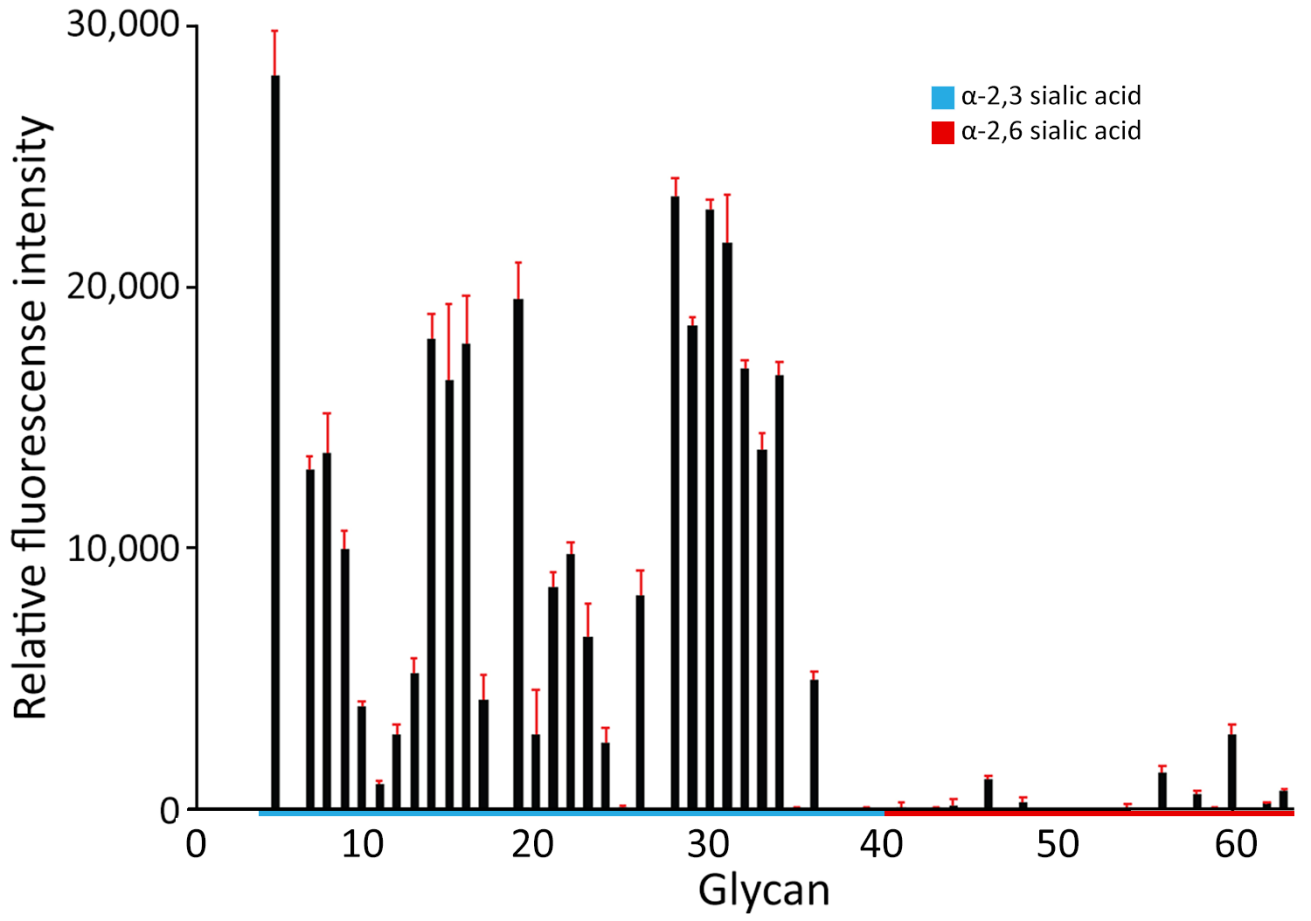
Glycan microarray analysis of highly pathogenic avian influenza A(H5N1) clade 2.3.4.4b virus isolated from poultry farm workers, Washington, USA, 2024. Clade 2.3.4.4b genotype D1.1 H5N1 virus A/Washington/240/2024 was isolated from a confirmed Washington human case. Glycans containing α-2,3 sialic acid and α-2,6 sialic acid are shown. Error bars reflect SDs from 6 independent replicates on the microarray. Details of the glycan structures are listed in Appendix Table 2.

The HA sequences from the Washington D1.1 viruses also lacked changes previously associated with increased binding to mammalian-like α2,6 sialic acid receptors. Glycan microarray analysis of the representative virus isolate A/Washington/240/2024 suggested that the Washington D1.1 H5N1 virus retained preferential binding to avian-like α2,3 sialic acid receptors (Figure 2). We used 3 A(H5) clade 2.3.4.4b prepandemic CVVs in this study: IDCDC‐RG78A (A/American wigeon/South Carolina/22-000345-001/2021), NIID-002 (A/Ezo red fox/Hokkaido/1/2022), and IDCDC-RG71A (A/Astrakhan/3212/2020). Compared with the most closely related CVV, NIID-002 (HA group ea3), the HA sequences from Washington H5N1 D1.1 viruses (also HA group ea3) all carried 2 amino acid differences at T36A and N476D (mature H5 numbering); neither substitution was located within putative antigenic sites. The HA from Washington H5N1 D1.1 viruses also had 3 amino acid differences relative to IDCDC-RG71A (HA group ea3) and 6 relative to IDCDC‐RG78A (HA group ea1), and 1 difference was in the putative antigenic site D (Appendix Table 3). Hemagglutination inhibition (HI) assays indicated that all available Washington H5N1 D1.1 virus isolates cross-reacted well with ferret antisera raised against each of the 3 clade 2.3.4.4b CVVs. Ferret antiserum raised to NIID-002 and IDCDC‐RG78A cross-reacted with the D1.1 viruses at heterologous HI titers equal to or within 2-fold of the homologous HI titer (Table). Ferret antisera raised against IDCDC-RG71A cross-reacted with the D1.1 viruses at heterologous HI titers 2- to 4-fold lower than the homologous HI titer. Ferret antiserum raised against A/Texas/37/2024, the virus isolated from an H5N1 human case in 2024 associated with a dairy cattle outbreak (genotype B3.13), also cross-reacted well with the Washington D1.1 H5N1 virus isolates at heterologous HI titers <2-fold of the homologous HI titer (Table).

## Conclusions

We detected human cases of HPAI H5N1 clade 2.3.4.4b genotype D1.1 in poultry farm workers in Washington. Additional human cases of H5N1 D1.1 virus infection have been subsequently reported, including a fatal human case detected in Louisiana, USA (*13*), and 2 severe human cases, 1 detected in British Columbia, Canada (*14*), and 1 in Wyoming, USA (*15*). Among the virus genomes detected in the Washington cases, we noted no changes that are known to be associated with mammalian adaptation, increased infectivity, or transmissibility among humans. Washington H5N1 D1.1 virus retained avian-like α2,3 sialic acid receptor binding preference and cross-reacted well with ferret antiserum raised against A(H5) clade 2.3.4.4b prepandemic CVVs available to vaccine manufacturers. Nonetheless, continued surveillance and testing of clade 2.3.4.4b A(H5) viruses remain essential for influenza pandemic preparedness.

**Appendix.** Additional information on highly pathogenic avian influenza A(H5N1) clade 2.3.4.4b virus infection in poultry farm workers, Washington, USA, 2024.
