# Supplementary material for: Highly Pathogenic Avian Influenza A(H5N1) Clade 2.3.4.4b Virus Infection in Poultry Farm Workers, Washington, USA, 2024
Source: Emerg Infect Dis. 2025 Dec;31(12):2297–301. doi: 10.3201/eid3112.251118 (PMC12782260; doi:10.3201/eid3112.251118)
Supplement: Supplementary file 1 — Appendix. Additional information on highly pathogenic avian influenza A(H5N1) clade 2.3.4.4b virus infection in poultry farm workers, Washington, USA, 2024. [file 25-1118-Techapp-s1.pdf]

Article DOI: https://doi.org/10.3201/eid3112.251118

EID cannot ensure accessibility for supplementary materials supplied by authors. Readers who have difficulty accessing supplementary content should contact the authors for assistance.

Highly Pathogenic Avian Influenza A(H5N1) Clade 2.3.4.4b Virus Infection in Poultry Farm Workers, Washington, USA, 2024

Appendix

Methods

Influenza A Confirmation and Sequencing of the Clinical Specimens

Washington State Public Health Laboratory performed the initial real-time RT-PCR testing of the clinical specimens collected from symptomatic poultry farm workers with exposure to A(H5N1) virus-infected poultry. The median days between symptoms onset and sample collection were 2 days (range, 0 to 4 days). Conjunctival or nasopharyngeal specimens were then shipped to the Centers for Disease Control and Prevention (CDC, Atlanta, GA, United States) for confirmatory testing. To isolate nucleic acids for confirmatory testing, viral subtyping, and sequencing, RNA was extracted from 120 µL of clinical specimen using an EZ1 DSP Virus Kit (QIAGEN, Hilden, Germany) following the manufacturer's instruction. The purified RNA was tested for the detection of universal influenza A matrix gene and A(H5) HA gene targets using a TaqMan real-time RT-PCR assay (*1*). Influenza A viral genome was amplified from the extracted RNA using multi-segment RT-PCR with universal influenza A primers (*2*). The resulting amplicons were subjected to library preparation using the DNA Prep library preparation kit (Illumina) and sequenced on the Illumina MiSeq platform using the MiSeq v2 300 cycle kit (Illumina) following the manufacturer's protocol. The output reads were analyzed using the iterative refinement meta-assembler (IRMA) pipeline (*3*). Assembled Influenza A viral genome segments that met quality thresholds (100X minimum average coverage and 25X minimum coverage at each nucleotide except for the 5% region at the 5′ and 3′ ends) were submitted to

both the GISAID (https://gisaid.org) and GenBank (https://www.ncbi.nlm.nih.gov/genbank) databases (Appendix Table 1).

Virus Isolation

For all clinical specimens that were confirmed A(H5)-positive by the CDC, virus isolation was attempted in embryonated chicken eggs. For select A(H5)-positive specimens with enough volume, virus isolation was also attempted in Madin Darby Canine Kidney (MDCK) cell lines. Inoculated eggs and cells were incubated at 37°C for 24–48 hours. Afterward, egg allantoic fluid or cell culture supernatant was harvested, and hemagglutination titer was determined using turkey red blood cells (RBCs, Lampire Biologic Laboratories, Pipersville, PA, USA). Stocks of successfully isolated viruses were sequenced as previously described and stored in a −80°C freezer.

Antigenic Characterization

Hemagglutination inhibition (HI) tests were conducted following standard protocols (*4*). Briefly, test ferret antisera were pre-treated with receptor-destroying enzyme (RDE, Denka Seiken, Tokyo, Japan) and adsorbed with turkey RBCs. Then, ferret antisera were pipetted into a 96-well V-bottom plate followed by 2-fold serial dilutions. Each antisera dilution (25 µL) was subsequently mixed with 25 µL of test antigen virus standardized to 8 hemagglutination units (HAU)/50 µL in phosphate-buffered saline (PBS). Following a 30-minute incubation at room temperature, 50 µL turkey RBCs in PBS ($4.0 \pm 0.4 \times 10^7$ cells/mL) were added and incubated for another 30 minutes to allow the RBCs to settle. The HI titer was determined as the reciprocal of the highest dilution of ferret antisera that inhibited hemagglutination.

Phylogenetic Analysis

A phylogenetic tree was built for each available gene segment (codon complete or near complete) of D1.1 human cases after alignment with closely related virus sequences obtained from GISAID (https://platform.gisaid.org) and the Short Read Archive/NCBI. Sequences for each virus strain were aligned using BioEdit v7.0 and the MUSCLE algorithm (*5*). Neighbor-joining phylogenetic trees were built using MEGA7.0 software with 1,000 bootstraps and the Juke-Cantor Model of evolution with uniform rates (https://www.megasoftware.net).

Glycan Microarray Analysis

Glycan microarray slides used in this study were produced under contract for the Centers for Disease Control and Prevention by James Paulson at The Scripps Research Institute (La Jolla, CA). Glycans present on the array are listed in Appendix Table 2). β-Propiolactone (BPL)-inactivated D1.1 viruses were diluted in PBS with 2% bovine serum albumin to a hemagglutination titer of 128. The diluted virus suspension was applied to and incubated on the glycan microarrays on ice for 1.5 hours with gentle rotation, after which slides were washed with PBS containing 0.05% Tween 20 (PBS-T) followed by PBS only. This wash process followed each incubation step. As primary antibody, ferret antiserum raised against IDCDC-RG80A (A/chicken/Ghana/AVL-763_21VIR7050–39/2021) pre-diluted at 1:500 in PBS, was incubated on the arrays for 30 minutes. Slides were washed, then followed by another 30-minute incubation with a secondary biotin-labeled mouse anti-ferret IgG antibody (Rockland Inc.) using a 1:200 dilution in PBS. After washing, a final 30-minute incubation step with Streptavidin Alexa Fluor 488 Conjugated diluted in PBS at 1:2000 dilution (Thermo Fisher Scientific, MA, USA) was performed. Slides were washed sequentially with PBS-T, PBS and H_2O, air dried, and then scanned for fluorescence using an Innoscan 1100AL fluorescence scanner (Innopsys, Carbonne, France) using the 488 nm laser. Spot intensities were quantified and analyzed using the system's Mapix data acquisition and analysis software.

References

1. Centers for Disease Control and Prevention. CDC laboratory support for influenza surveillance (CLSIS) SharePoint site [cited 2025 Sep 24]. https://www.cdc.gov/flu-pep-panel/clsis/?CDC_AAref_Val=https://www.cdc.gov/flu/clsis/index.htm

2. Zhou B, Donnelly ME, Scholes DT, St George K, Hatta M, Kawaoka Y, et al. Single-reaction genomic amplification accelerates sequencing and vaccine production for classical and swine origin human influenza A viruses. J Virol. 2009;83:10309–13. PubMed https://doi.org/10.1128/JVI.01109-09

3. Shepard SS, Meno S, Bahl J, Wilson MM, Barnes J, Neuhaus E. Viral deep sequencing needs an adaptive approach: IRMA, the iterative refinement meta-assembler. BMC Genomics. 2016;17:708. PubMed https://doi.org/10.1186/s12864-016-3030-6

4. World Health Organization. Manual for the laboratory diagnosis and virological surveillance of influenza [cited 2025 Sep 24]. https://iris.who.int/bitstream/handle/10665/44518/9789241548090_eng.pdf

5. Edgar RC. MUSCLE: multiple sequence alignment with high accuracy and high throughput. Nucleic Acids Res. 2004;32:1792–7. PubMed https://doi.org/10.1093/nar/gkh340

Appendix Table 1. GISAID and NCBI submission numbers for Washington D1.1 A(H5N1) viruses.

Strain name	Passage Information	GISAID EPI ISL	NCBI HA
A/Washington/239/2024	Original	EPI_ISL_19512045	PQ525413
A/Washington/239/2024	C1	EPI_ISL_19531298	PQ573554
A/Washington/240/2024	Original	EPI_ISL_19512046	PQ525416
A/Washington/240/2024	E1	EPI_ISL_19531299	PQ573562
A/Washington/247/2024	Original	EPI_ISL_19512047	PQ525420
A/Washington/254/2024	Original	EPI_ISL_19531303	PQ615328
A/Washington/254/2024	E1	EPI_ISL_19666173	PQ885521
A/Washington/255/2024	Original	EPI_ISL_19552697	PQ615366

Appendix Table 2. Glycan structures presented on the microarray slide

Chart no.	Structure	Description
1	α-Neu5Ac-Sp8	α-Neu5Ac
2	α-Neu5Ac-Sp11	α-Neu5Ac
3	β-Neu5Ac-Sp8	β-Neu5Ac
Glycans that contain α-2,3 sialic acid		
4	Neu5Acα2-3(6-O-Su)Galβ1-4(Fucα1-3)GlcNAcβ-Sp8	α2-3 so4
5	Neu5Acα2-3Galβ1-3(6OSO3)GalNAcα-Sp8	α2-3 so4
6	Neu5Acα2-3Galβ1-4(6OSO3)GlcNAcβ-Sp8	α2-3 so4
7	Neu5Acα2-3Galβ1-4(Fucα1-3)(6OSO3)GlcNAcβ-Sp8	α2-3 so4
8	Neu5Acα2-3Galβ1-3(6OSO3)GlcNAc-Sp8	α2-3 so4
9	Neu5Acα2-3Galβ1-3(Neu5Acα2-3Galβ1-4)GlcNAcβ-Sp8	di-sialoside
10	Neu5Acα2-3Galβ1-3(Neu5Acα2-3Galβ1-4GlcNAcβ1-6)GalNAc-Sp14	di-sialoside
11	Neu5Acα2-3Galβ1-4GlcNAcβ1-2Manα1-3(Neu5Acα2-3Galβ1-4GlcNAcβ12Manα1-6)Manβ1-4GlcNAcβ1-4GlcNAcβ-Sp12	α2-3 biantennary
12	Neu5Acα2-3Galβ-Sp8	α2-3
13	Neu5Acα2-3GalNAcα-Sp8	α2-3
14	Neu5Acα2-3Galβ1-3GalNAcα-Sp8	α2-3
15	Neu5Acα2-3Galβ1-3GlcNAcβ-Sp0	α2-3
16	Neu5Acα2-3Galβ1-3GlcNAcβ-Sp8	α2-3
17	Neu5Acα2-3Galβ1-4Glcβ-Sp0	α2-3
18	Neu5Acα2-3Galβ1-4Glcβ-Sp8	α2-3
19	Neu5Acα2-3Galβ1-4GlcNAcβ-Sp0	α2-3
20	Neu5Acα2-3Galβ1-4GlcNAcβ-Sp8	α2-3
21	Neu5Acα2-3GalNAcβ1-4GlcNAcβ-Sp0	α2-3
22	Neu5Acα2-3Galβ1-4GlcNAcβ1-3Galβ1-4GlcNAcβ-Sp0	α2-3
23	Neu5Acα2-3Galβ1-3GlcNAcβ1-3Galβ1-3GlcNAcβ-Sp0	α2-3
24	Neu5Acα2-3Galβ1-4GlcNAcβ1-3Galβ1-4GlcNAcβ1-3Galβ1-4GlcNAcβ- Sp0	α2-3
25	Neu5Acα2-3Galβ1-4GlcNAcβ1-3Galβ1-3GlcNAcβ-Sp0	α2-3
26	Neu5Acα2-3Galβ1-3GalNAc-Sp14	α2-3
27	Neu5Acα2-3Galβ1-4(Fucα1-3)GlcNAcβ1-6(Galβ1-3)GalNAc-Sp14	α2-3 fucosylated
28	Neu5Acα2-3Galβ1-3(Fucα1-4)GlcNAcβ-Sp8	α2-3 fucosylated
29	Neu5Acα2-3Galβ1-4(Fucα1-3)GlcNAcβ-Sp0	α2-3 fucosylated
30	Neu5Acα2-3Galβ1-4(Fucα1-3)GlcNAcβ-Sp8	α2-3 fucosylated
31	Neu5Acα2-3Galβ1-4(Fucα1-3)GlcNAcβ1-3Galβ-Sp8	α2-3 fucosylated
32	Neu5Acα2-3-galβ1-3(Fucα1-4)GlcNAcβ1-3Galβ1-4(Fucα1-3)GlcNAcβ-Sp0	α2-3 fucosylated
33	Neu5Acα2-3Galβ1-4(Fucα1-3)GlcNAcβ1-3Galβ1-4(Fuca1-3)GlcNAcβ-Sp0	α2-3 fucosylated
34	Neu5Aca2-3Galβ1-4(Fuca1-3)GlcNAcβ1-3Galβ1-4(Fuca1-3)GlcNAcβ1-3Galβ1-4(Fuca1-3)GlcNAcβ-Sp0	α2-3 fucosylated
35	Neu5Acα2-3(GalNAcβ1-4)Galβ1-4GlcNAcβ-Sp0	α2-3 internal
36	Neu5Acα2-3(GalNAcβ1-4)Galβ1-4GlcNAcβ-Sp8	α2-3 internal
37	Neu5Acα2-3(GalNAcβ1-4)Galβ1-4Glcβ-Sp0	α2-3 internal
38	Neu5Acα2-3(Galβ1-3GalNAcβ1-4)Galβ1-4Glcβ-Sp0	α2-3 internal

Chart no.	Structure	Description
39	Neu5Acα2-3(Fucα1-2Galβ1-3GalNAcβ1-4)Galβ1-4Glcβ-Sp0	α2-3 internal
40	Neu5Acα2-3(Fucα1-2Galβ1-3GalNAcβ1-4)Galβ1-4Glcβ-Sp9	α2-3 internal
Glycans that contain α-2,6 sialic acid		
41	Neu5Acα2-6Galβ1-4[6OSO3]GlcNAcβ-Sp8	α2-6 so4
42	Neu5Acα2-6Galβ1-4GlcNAcβ1-2Manα16(Galβ1-4GlcNAcβ1-2Manα1-3)Manβ1-4GlcNAcβ1-4GlcNAcβ-Sp12	α2–6 branched
43	Neu5Acα2-6Galβ1-4GlcNAcβ1-2Manα1-3(Neu5Acα2-6Galβ1-4GlcNAcβ1-2Manα1-6)Manβ1-4GlcNAcβ1-4GlcNAcβ-Sp12	α2-6 biantenary
44	NeuAcα(2-6)-Galβ(1-4)-GlcNAcβ(1-3)-Galβ(1-4)-GlcNAcβ(1-2)-Manα(1-3)-[NeuAcα(2-6)-Galβ(1-4)-GlcNAcβ(1-3)-Galβ(1-4)-GlcNAcβ(1-2)-Manα(1-6)]-Manβ(1-4)-GlcNAcβ(1-4)-GlcNAcβ-Sp12	α2-6 biantenary
45	Neu5Acα2-6Galβ1-4GlcNAcβ1-3Galβ1-4GlcNAcβ1-3Galβ1-4GlcNAcβ1-2Manα1-3(Neu5Acα2-6Galβ1-4GlcNAcβ1-3Galβ1-4GlcNAcβ1-3Galβ1-4GlcNAcβ1-2Manα1-6)Manβ1-4GlcNAcβ1-4GlcNAcβ-Sp12	α2-6 biantenary
46	Neu5Acα2-6Galβ1-4GlcNAcβ1-3Galβ1-4GlcNAcβ(1-3)(Neu5Acα2-6Galβ1-4GlcNAcβ1-3Galβ1-4GlcNAcβ1-6)GalNAca-Sp14	α2-6 biantenary
47	Neu5Acα2-6Galβ1-4GlcNAcβ1-2Manα1-3(Neu5Acα2-6Galβ1-4GlcNAcβ12Manα1-6)Manβ1-4GlcNAcβ1-4GlcNAcβ-Sp8	α2-6 biantenary
48	Neu5Acα2-6Galβ1-4GlcNAcβ1-2Manα1-3(Neu5Acα2-6Galβ1-4GlcNAcβ12Manα1-6)Manβ1-4GlcNAcβ1-4GlcNAcβ-Sp12	α2-6 biantenary
49	Neu5Acα2-6Galβ1-4GlcNAcβ1-2Manα1-3(Galβ1-4GlcNAcβ1-2Manα16)Manβ1-4GlcNAcβ1-4GlcNAcβ-Sp12	α2-6 biantenary
50	Neu5Acα2-6GalNAcα-Sp8	α2-6
51	Neu5Acα2-6Galβ-Sp8	α2-6
52	Neu5Acα2-6Galβ1-4Glcβ-Sp8	α2-6
53	Neu5Acα2-6Galβ1-4GlcNAcβ-Sp0	α2-6
54	Neu5Acα2-6Galβ1-4GlcNAcβ-Sp8	α2-6
55	Neu5Acα2-6GalNAcβ1-4GlcNAcβ-Sp0	α2-6
56	Neu5Acα2-6Galβ1-4GlcNAcβ1-3Galβ1-4GlcNAcβ-Sp0	α2-6
57	Neu5Acα2-6Galβ1-4GlcNAcβ1-3Galβ1-4GlcNAcβ1-3GalNAca-Sp14	α2-6
58	Neu5Aca2-6Galβ1-4GlcNAcβ1-3Galβ1-4GlcNAcβ1-3Galβ1-4GlcNAcβ-Sp0	α2-6
59	Neu5Acα2-6Galβ1-4GlcNAcβ1-3Galβ1-4(Fucα1-3)GlcNAcβ1-3Galβ1-4(Fucα1-3)GlcNAcβ-Sp0	α2-6 fucosylated
60	Neu5Acα2-6(Galβ1-3)GlcNAcβ1-4Galβ1-4Glcβ-Sp10	α2-6 internal
61	Neu5Acα2-6(Galβ1-3)GalNAca-Sp14	α2-6 internal
62	Neu5Acα2-6Galβ1-4GlcNAcβ1-6(Galβ1-3)GalNAca-Sp14	α2-6 internal
63	NeuAcα2-6Galβ1-4GlcNAcβ1-3Galβ1-4GlcNAcβ1-6(Galβ(1-3)GalNAca-Sp14	α2-6 internal

Appendix Table 3. Washington D1.1 A(H5N1) HA amino acid changes relative to 2.3.4.4b A(H5) CVVs. The Washington D1.1 A(H5N1) viruses are shown in green and the pre-pandemic CVVs in red.

Mature H5 numbering	A/Ezo red fox/Hokkaido/1/2022	A/American Wigeon/South Carolina/22-000345-001/2021	A/Astrakhan/3212/2020_H5N8	A/Washington/239/2024	A/Washington/240/2024	A/Washington/247/2024	A/Washington/254/2024	A/Washington/255/2024	Annotation
36	T			A	A	A	A	A	
104	L	M							
140	A								Antigenic site A
186	E								Antigenic site B
210	V	A							Antigenic site D
222	Q								Antigenic site D
325	R	K	K						
476	N			D	D	D	D	D	
511	I	V							
No. amino acid changes		4	1	2	2	2	2	2	vs. A/Ezo red fox/Hokkaido/1/2022
			3	6	6	6	6	6	vs. A/American Wigeon/South Carolina/22-000345-001/2021
				3	3	3	3	3	vs. A/Astrakhan/3212/2020_H5N8

A

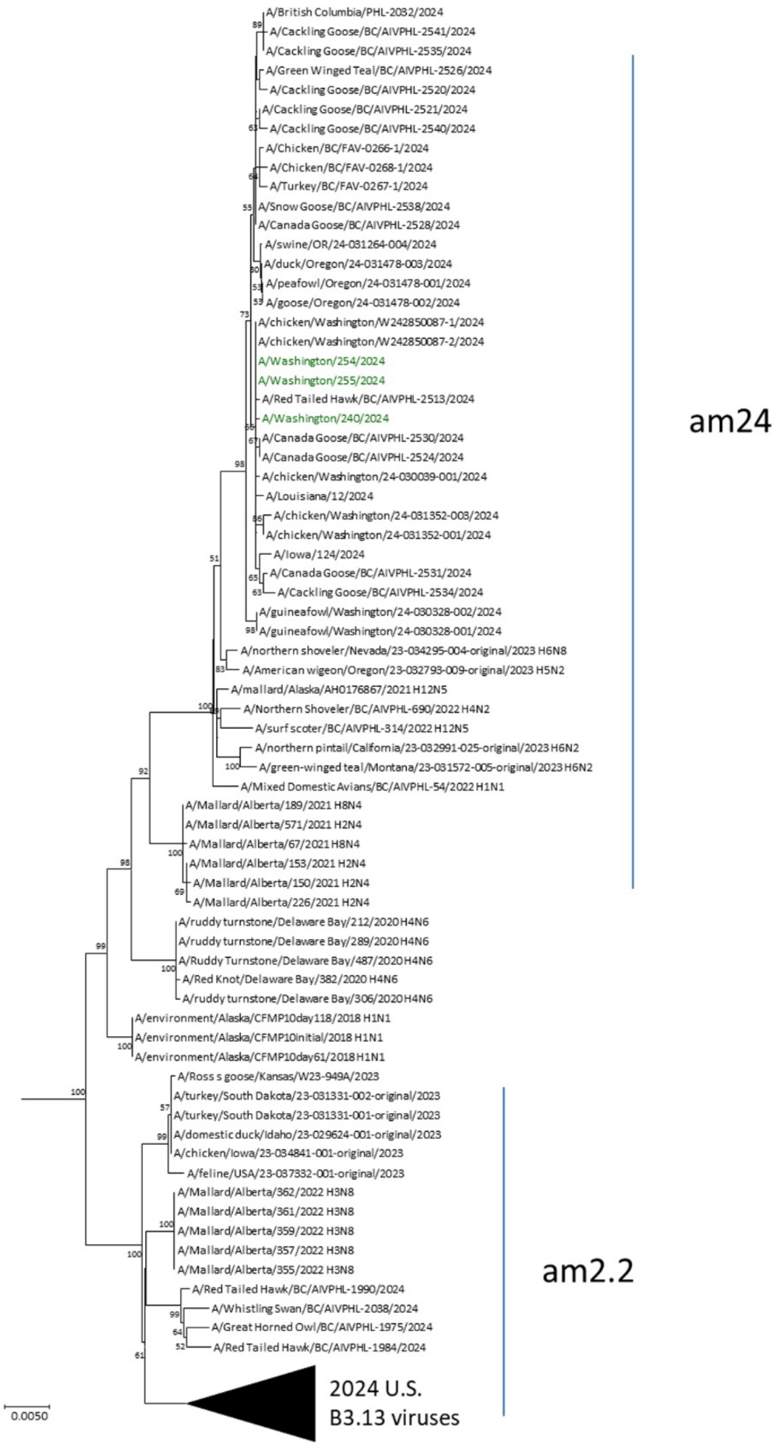

Appendix Figure 1. Neighbor-joining phylogenetic trees of the PB2 gene segment. The Washington D1.1 A(H5N1) viruses are shown in green and the pre-pandemic CVVs in red. Bootstrap values >50 (generated from 1,000 replicates) are labeled on branch nodes. Genetic groups are labeled on the tree. Scale bar indicates nucleotide substitutions per site.

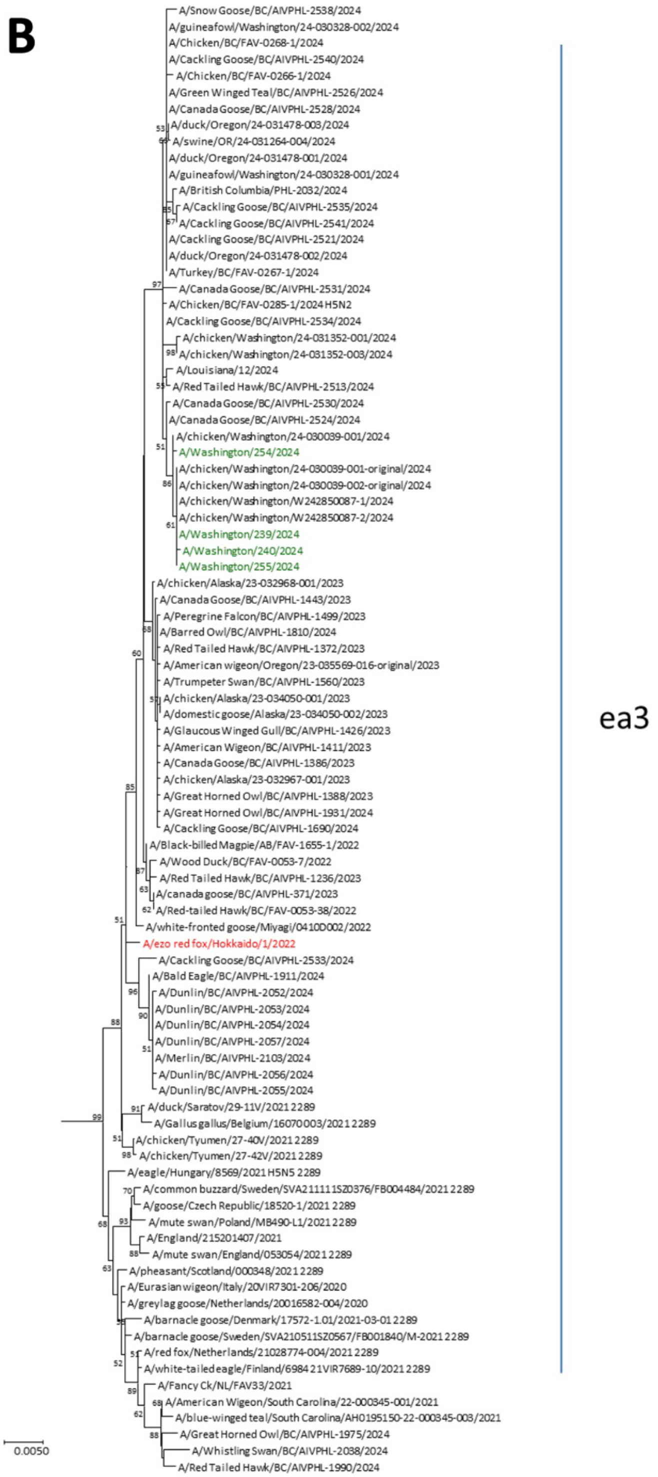

Appendix Figure 2. Neighbor-joining phylogenetic trees of the PB1 gene segment. The Washington D1.1 A(H5N1) viruses are shown in green and the pre-pandemic CVVs in red. Bootstrap values >50 (generated from 1,000 replicates) are labeled on branch nodes. Genetic groups are labeled on the tree. Scale bar indicates nucleotide substitutions per site.

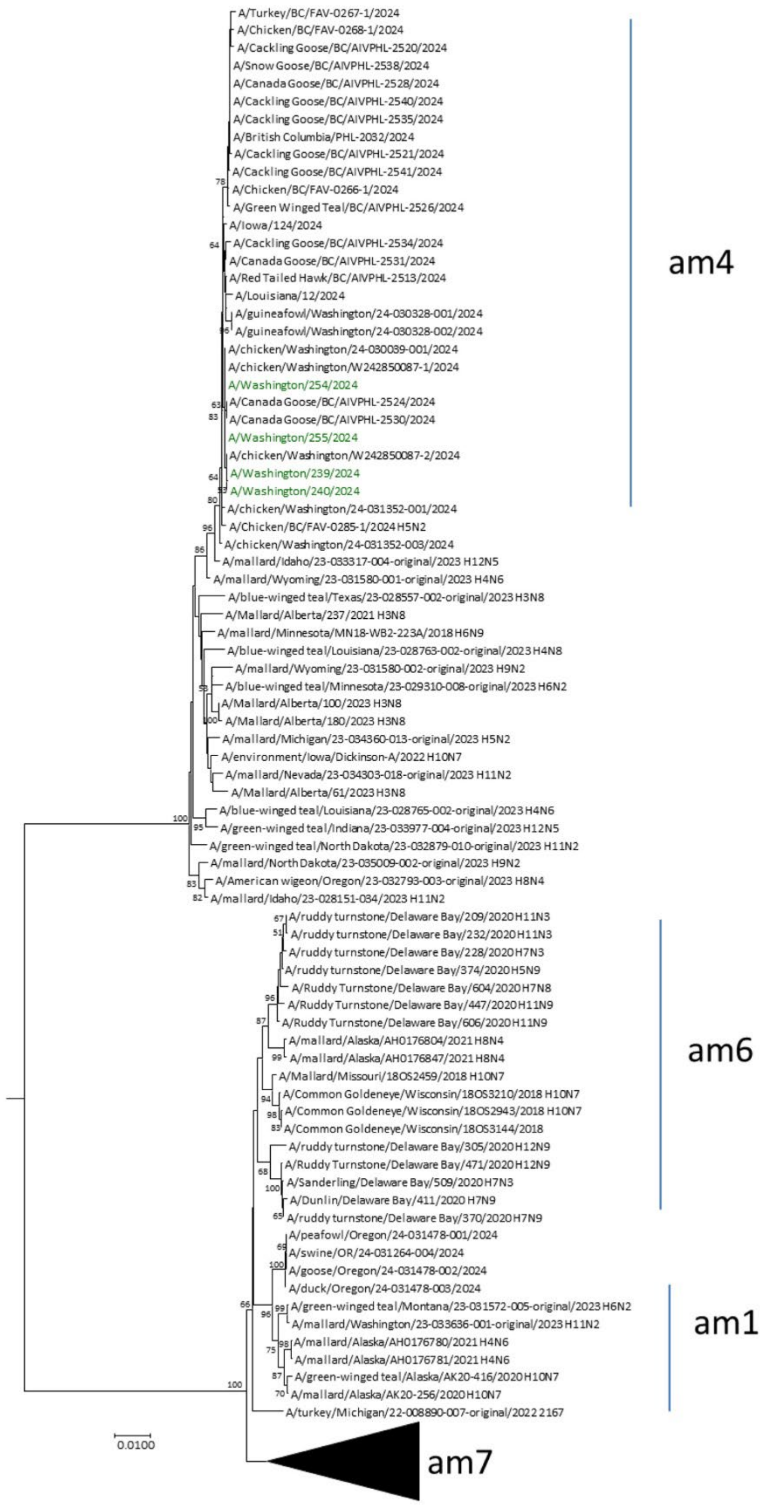

Appendix Figure 3. Neighbor-joining phylogenetic trees of the PA gene segment. The Washington D1.1 A(H5N1) viruses are shown in green and the pre-pandemic CVVs in red. Bootstrap values >50 (generated from 1,000 replicates) are labeled on branch nodes. Genetic groups are labeled on the tree. Scale bar indicates nucleotide substitutions per site.

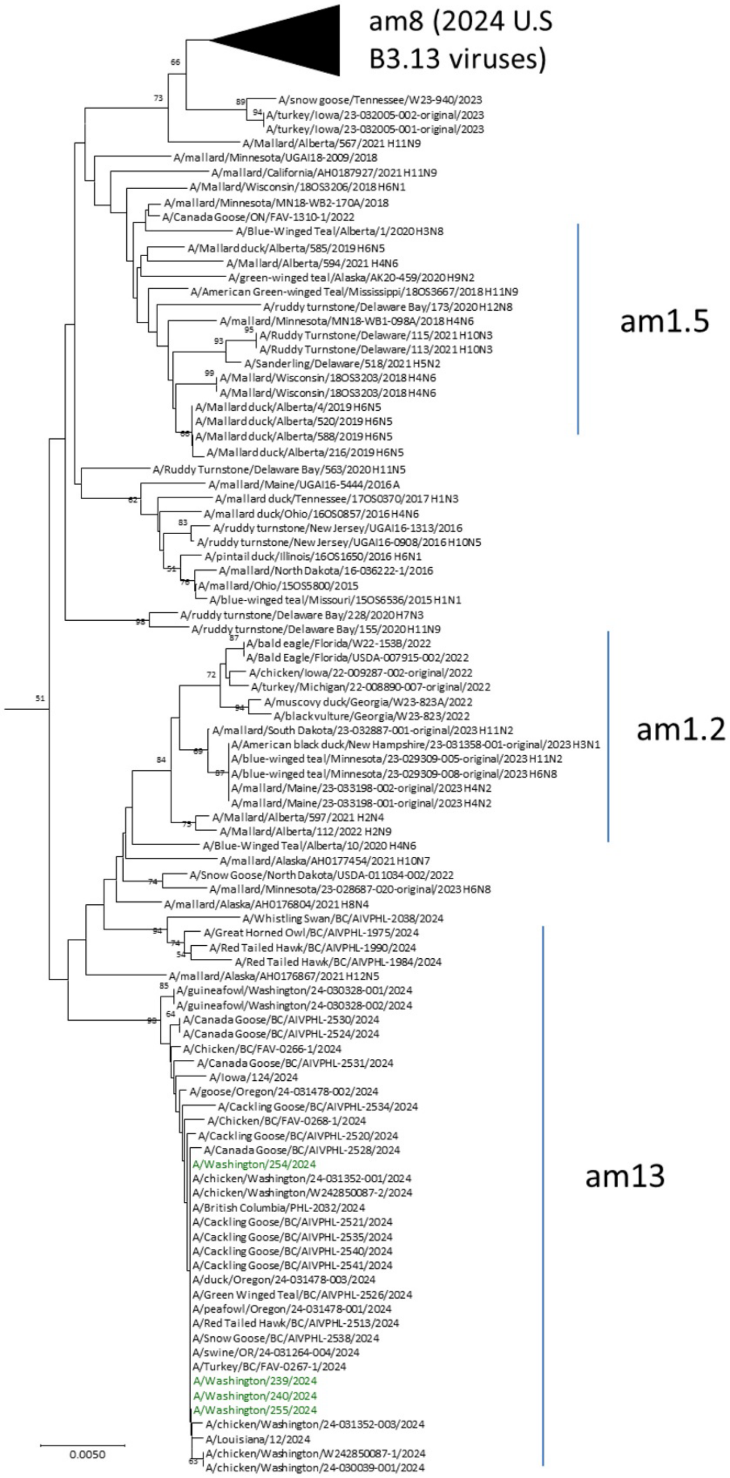

Appendix Figure 4. Neighbor-joining phylogenetic trees of the NP gene segment. The Washington D1.1 A(H5N1) viruses are shown in green and the pre-pandemic CVVs in red. Bootstrap values >50 (generated from 1,000 replicates) are labeled on branch nodes. Genetic groups are labeled on the tree. Scale bar indicates nucleotide substitutions per site.

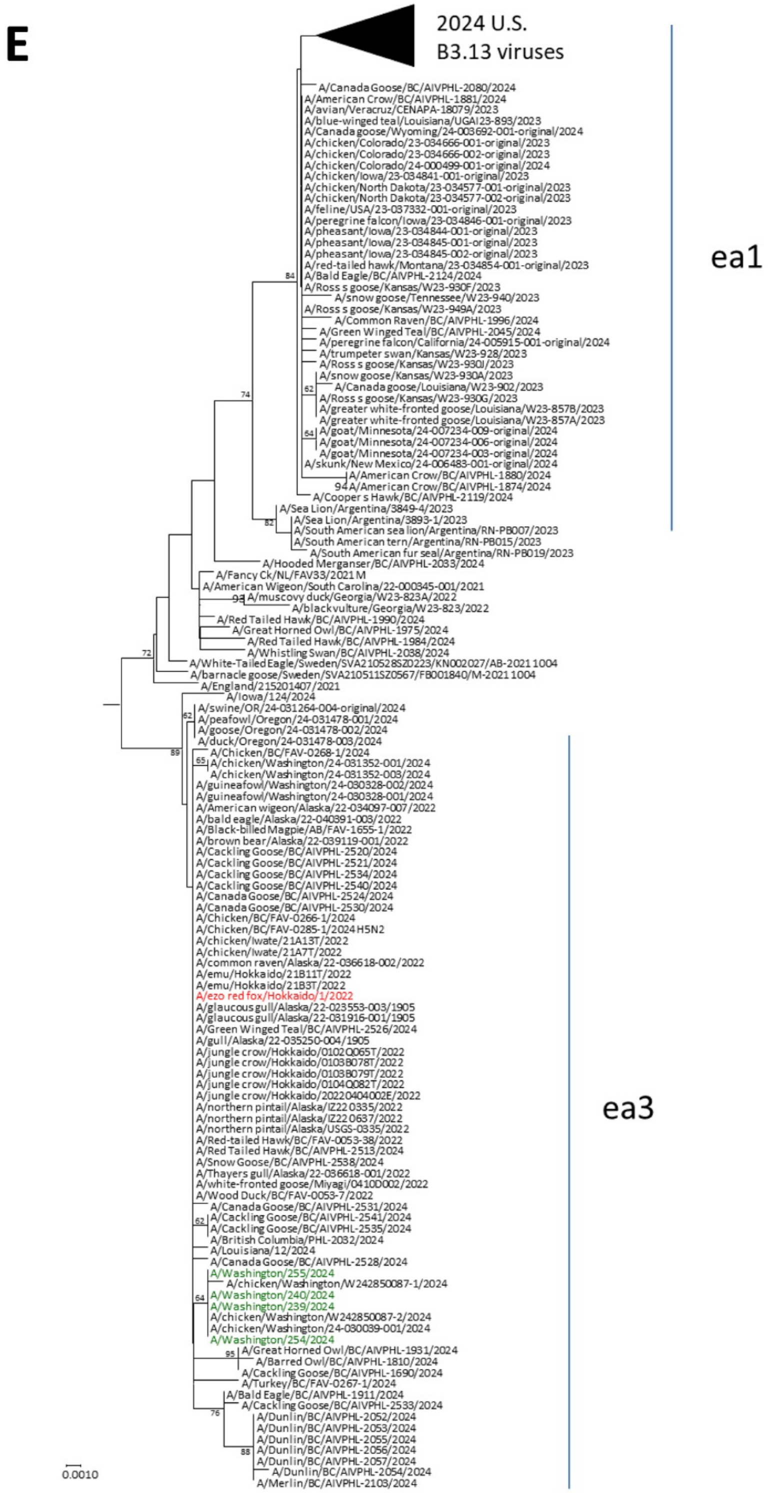

Appendix Figure 5. Neighbor-joining phylogenetic trees of the M gene segment. The Washington D1.1 A(H5N1) viruses are shown in green and the pre-pandemic CVVs in red. Bootstrap values >50 (generated from 1,000 replicates) are labeled on branch nodes. Genetic groups are labeled on the tree. Scale bar indicates nucleotide substitutions per site.

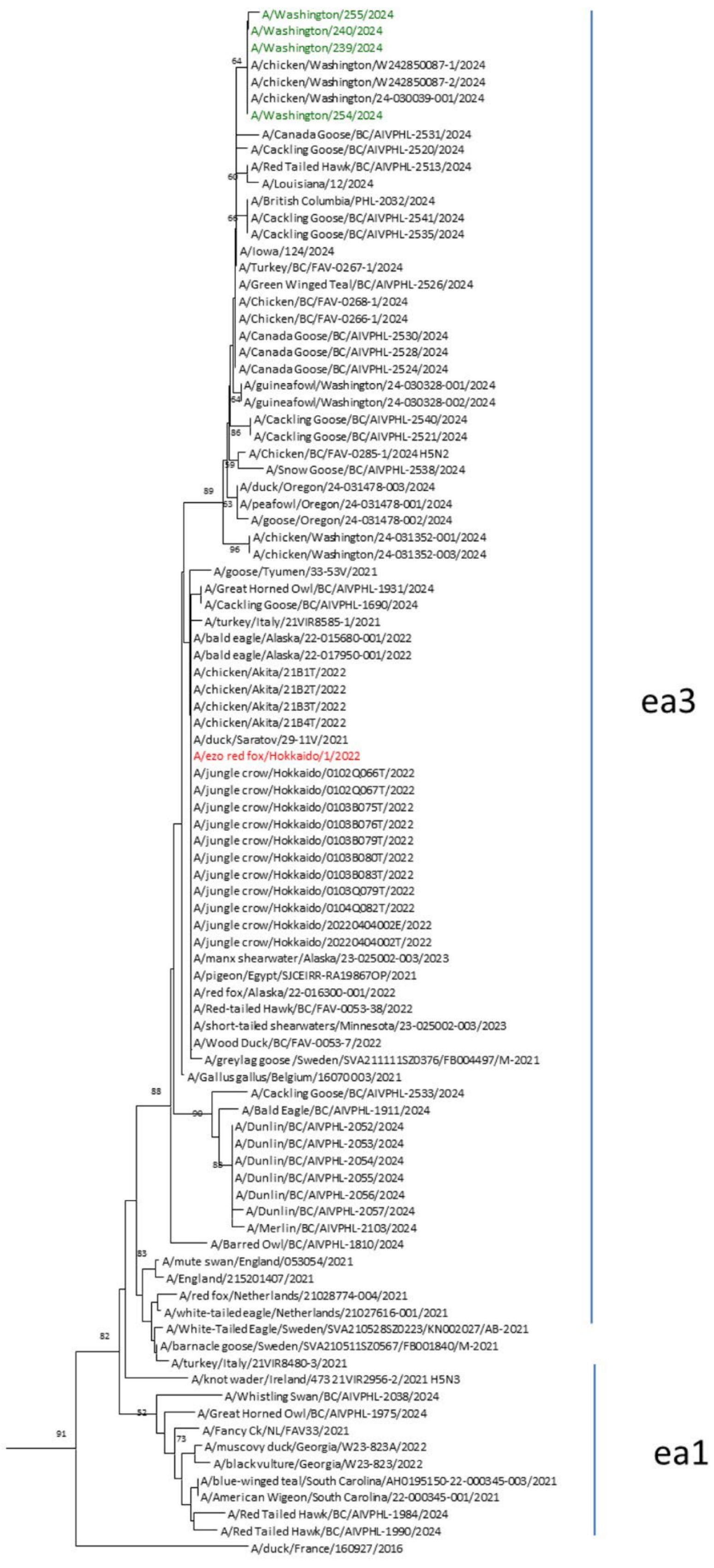
A/Washington/255/2024
A/Washington/240/2024
A/Washington/239/2024
A/chicken/Washington/W242850087-1/2024
A/chicken/Washington/W242850087-2/2024
A/chicken/Washington/24-030039-001/2024
A/Washington/254/2024
A/Canada Goose/BC/AIVPHL-2531/2024
A/Cackling Goose/BC/AIVPHL-2520/2024
A/Red Tailed Hawk/BC/AIVPHL-2513/2024
A/Louisiana/12/2024
A/British Columbia/PHL-2032/2024
A/Cackling Goose/BC/AIVPHL-2541/2024
A/Cackling Goose/BC/AIVPHL-2535/2024
A/Iowa/124/2024
A/Turkey/BC/FAV-0267-1/2024
A/Green Winged Teal/BC/AIVPHL-2526/2024
A/Chicken/BC/FAV-0268-1/2024
A/Chicken/BC/FAV-0266-1/2024
A/Canada Goose/BC/AIVPHL-2530/2024
A/Canada Goose/BC/AIVPHL-2528/2024
A/Canada Goose/BC/AIVPHL-2524/2024
A/guineafowl/Washington/24-030328-001/2024
A/guineafowl/Washington/24-030328-002/2024
A/Cackling Goose/BC/AIVPHL-2540/2024
A/Cackling Goose/BC/AIVPHL-2521/2024
A/Chicken/BC/FAV-0285-1/2024 H5N2
A/Snow Goose/BC/AIVPHL-2538/2024
A/duck/Oregon/24-031478-003/2024
A/peafowl/Oregon/24-031478-001/2024
A/goose/Oregon/24-031478-002/2024
A/chicken/Washington/24-031352-001/2024
A/chicken/Washington/24-031352-003/2024
A/goose/Tyumen/33-53V/2021
A/Great Horned Owl/BC/AIVPHL-1931/2024
A/Cackling Goose/BC/AIVPHL-1690/2024
A/turkey/Italy/21VIR8585-1/2021
A/bald eagle/Alaska/22-015680-001/2022
A/bald eagle/Alaska/22-017950-001/2022
A/chicken/Akita/21B1T/2022
A/chicken/Akita/21B2T/2022
A/chicken/Akita/21B3T/2022
A/chicken/Akita/21B4T/2022
A/duck/Saratov/29-11V/2021
A/ezo red fox/Hokkaido/1/2022
A/jungle crow/Hokkaido/0102Q066T/2022
A/jungle crow/Hokkaido/0102Q067T/2022
A/jungle crow/Hokkaido/0103B075T/2022
A/jungle crow/Hokkaido/0103B076T/2022
A/jungle crow/Hokkaido/0103B079T/2022
A/jungle crow/Hokkaido/0103B080T/2022
A/jungle crow/Hokkaido/0103B083T/2022
A/jungle crow/Hokkaido/0103Q079T/2022
A/jungle crow/Hokkaido/0104Q082T/2022
A/jungle crow/Hokkaido/20220404002E/2022
A/jungle crow/Hokkaido/20220404002T/2022
A/manx shearwater/Alaska/23-025002-003/2023
A/pigeon/Egypt/SJCEIRR-RA19867OP/2021
A/red fox/Alaska/22-016300-001/2022
A/Red-tailed Hawk/BC/FAV-0053-38/2022
A/short-tailed shearwaters/Minnesota/23-025002-003/2023
A/Wood Duck/BC/FAV-0053-7/2022
A/greylag goose /Sweden/SVA211111SZ0376/FB004497/M-2021
A/Gallus gallus/Belgium/16070003/2021
A/Cackling Goose/BC/AIVPHL-2533/2024
A/Bald Eagle/BC/AIVPHL-1911/2024
A/Dunlin/BC/AIVPHL-2052/2024
A/Dunlin/BC/AIVPHL-2053/2024
A/Dunlin/BC/AIVPHL-2054/2024
A/Dunlin/BC/AIVPHL-2055/2024
A/Dunlin/BC/AIVPHL-2056/2024
A/Dunlin/BC/AIVPHL-2057/2024
A/Merlin/BC/AIVPHL-2103/2024
A/Barred Owl/BC/AIVPHL-1810/2024
A/mute swan/England/053054/2021
A/England/215201407/2021
A/red fox/Netherlands/21028774-004/2021
A/white-tailed eagle/Netherlands/21027616-001/2021
A/White-Tailed Eagle/Sweden/SVA210528SZ0223/KN002027/AB-2021
A/barnacle goose/Sweden/SVA210511SZ0567/FB001840/M-2021
A/turkey/Italy/21VIR8480-3/2021
A/knot wader/Ireland/473 21VIR2956-2/2021 H5N3
A/Whistling Swan/BC/AIVPHL-2038/2024
A/Great Horned Owl/BC/AIVPHL-1975/2024
A/Fancy Ck/NL/FAV33/2021
A/muscovy duck/Georgia/W23-823A/2022
A/black vulture/Georgia/W23-823/2022
A/blue-winged teal/South Carolina/AH0195150-22-000345-003/2021
A/American Wigeon/South Carolina/22-000345-001/2021
A/Red Tailed Hawk/BC/AIVPHL-1984/2024
A/Red Tailed Hawk/BC/AIVPHL-1990/2024
A/duck/France/160927/2016
ea3
ea1
0.0050

Appendix Figure 6. Neighbor-joining phylogenetic trees of the NS gene segment. The Washington D1.1 A(H5N1) viruses are shown in green and the pre-pandemic CVVs in red. Bootstrap values >50 (generated from 1,000 replicates) are labeled on branch nodes. Genetic groups are labeled on the tree. Scale bar indicates nucleotide substitutions per site.
